# Supplementary material for: Fungal networks shape dynamics of bacterial dispersal and community assembly in cheese rind microbiomes
Source: Nat Commun. 2018 Jan 23;9:336. doi: 10.1038/s41467-017-02522-z (PMC5780524; doi:10.1038/s41467-017-02522-z)
Supplement: Supplementary file 1 — Supplementary Information [file 41467_2017_2522_MOESM1_ESM.pdf]

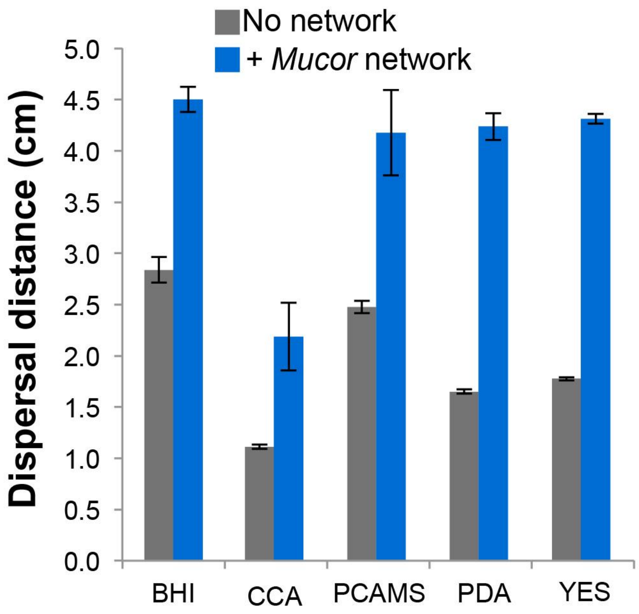

Supplementary Figure 1: Dispersal facilitation of *Serratia* by *Mucor* on different types of media. Distance travelled by *Serratia proteamaculans* BW106 across the surface of various types of media when grown with and without *Mucor lanceolatus* strain SN1 for 14 days. Other than the cheese curd agar, which was 1.7% agar, all other media were 1.5% agar. BHI = brain heart infusion agar (the medium used for most experiments throughout the study). CCA = cheese curd agar. PCAMS = plate count agar with milk and salt. PDA = potato dextrose agar. YES = yeast extract sucrose agar. Bars are mean distance from center of spot to bacterial colony edge (+/- one standard deviation, n = 4).

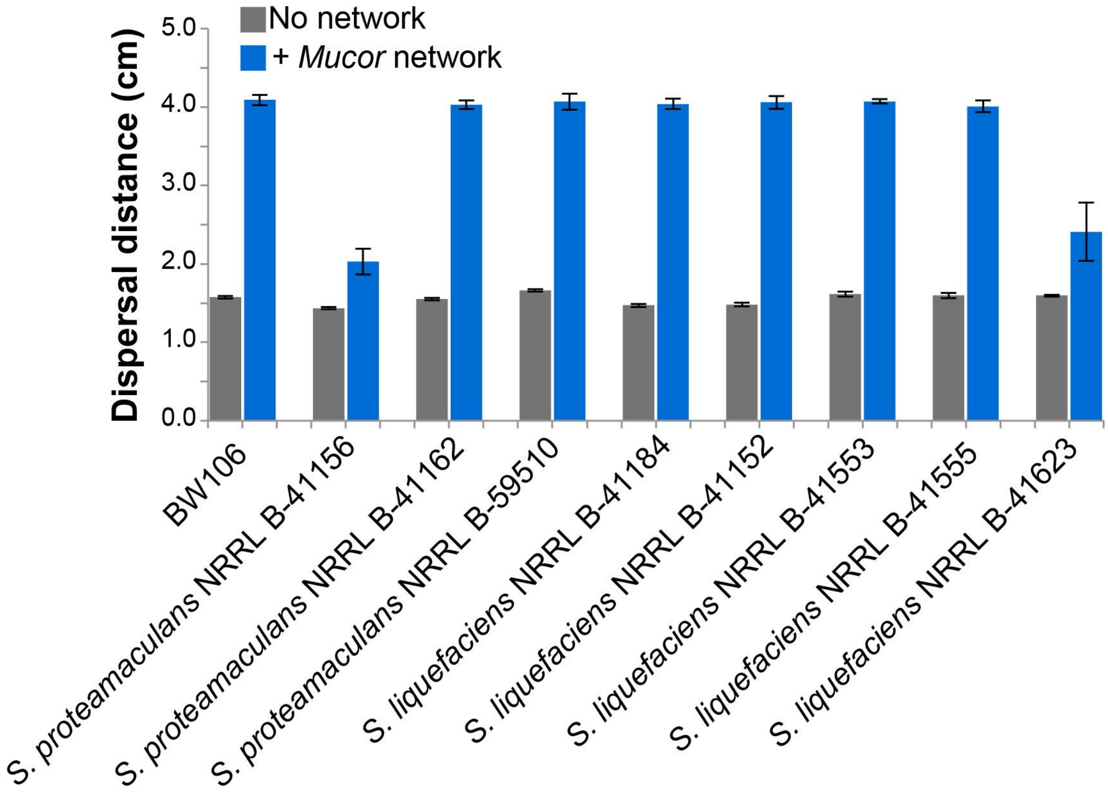

Supplementary Figure 2: Dispersal facilitation of different *Serratia* strains by *Mucor*. The co-spot assay used in Fig. 2a-b was also used to measure dispersal facilitation of a variety of closely related *Serratia* species (see phylogeny in Supplementary Figure 10). Bars are mean distance from center of spot to bacterial colony edge (+/- one standard deviation, n = 5).

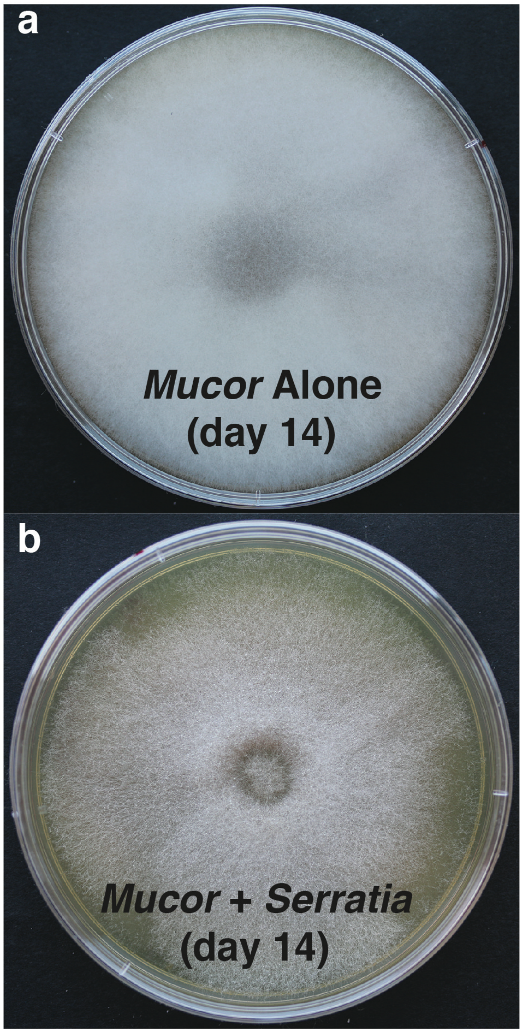

Supplementary Figure 3: Visual appearance of *Mucor* mycelium with and without *Serratia*. Representative plates from the co-culture growth assay, showing mycelium growth at day 14. **a** *Mucor* alone. **b** *Mucor* + *Serratia*. Petri dishes are 100 millimeters in diameter.

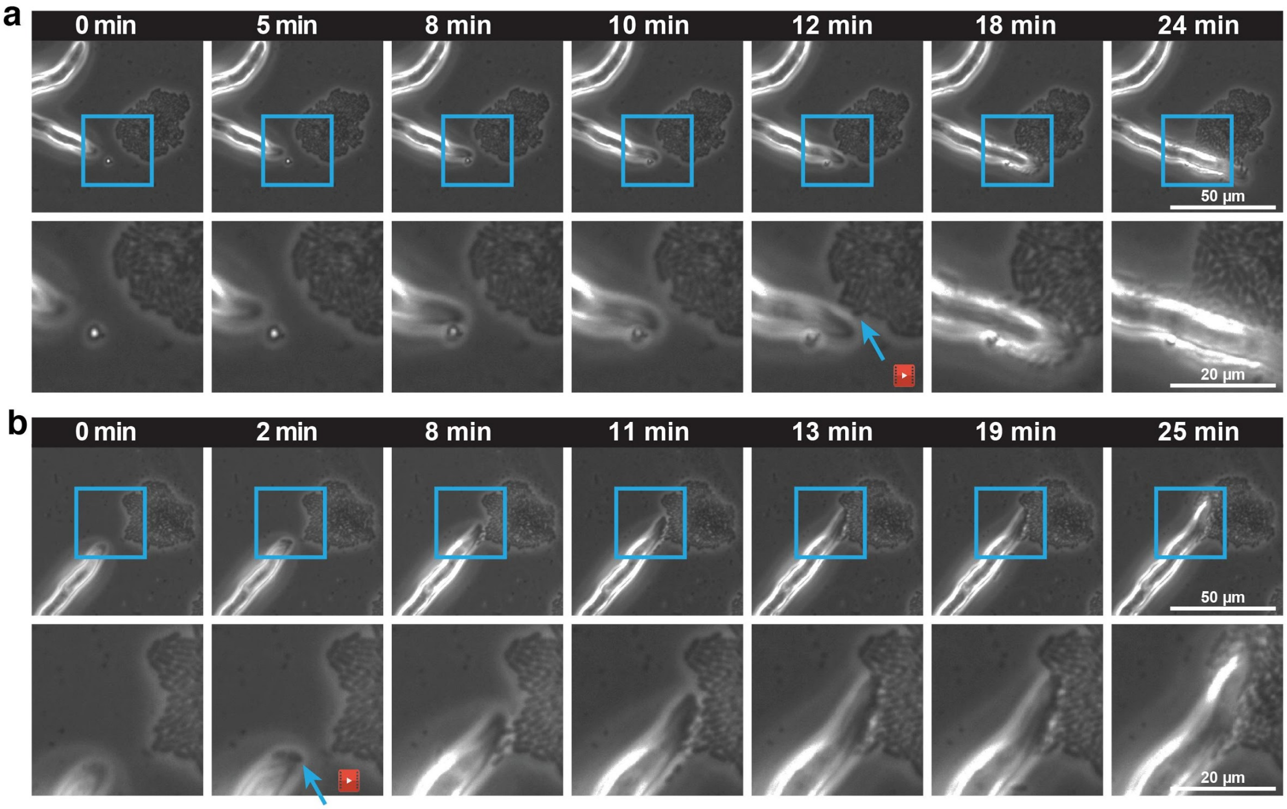

Supplementary Figure 4: Additional examples of *Serratia-Mucor* initial contacts. Each series (**a** and **b**) of *Serratia-Mucor* time-lapse photos is an independent colony contact imaged over 24 and 25 minutes, respectively. Blue box in top row indicates area that is magnified in row below. At the beginning, there is initial contact between a "pioneer" *Serratia* cell and the *Mucor* hyphae (blue arrows). Upon contact, the bacterium changes from sitting statically at the edge of the colony to rapid movement along the *Mucor* hyphae. Movie icon indicates image with corresponding supplementary movie.

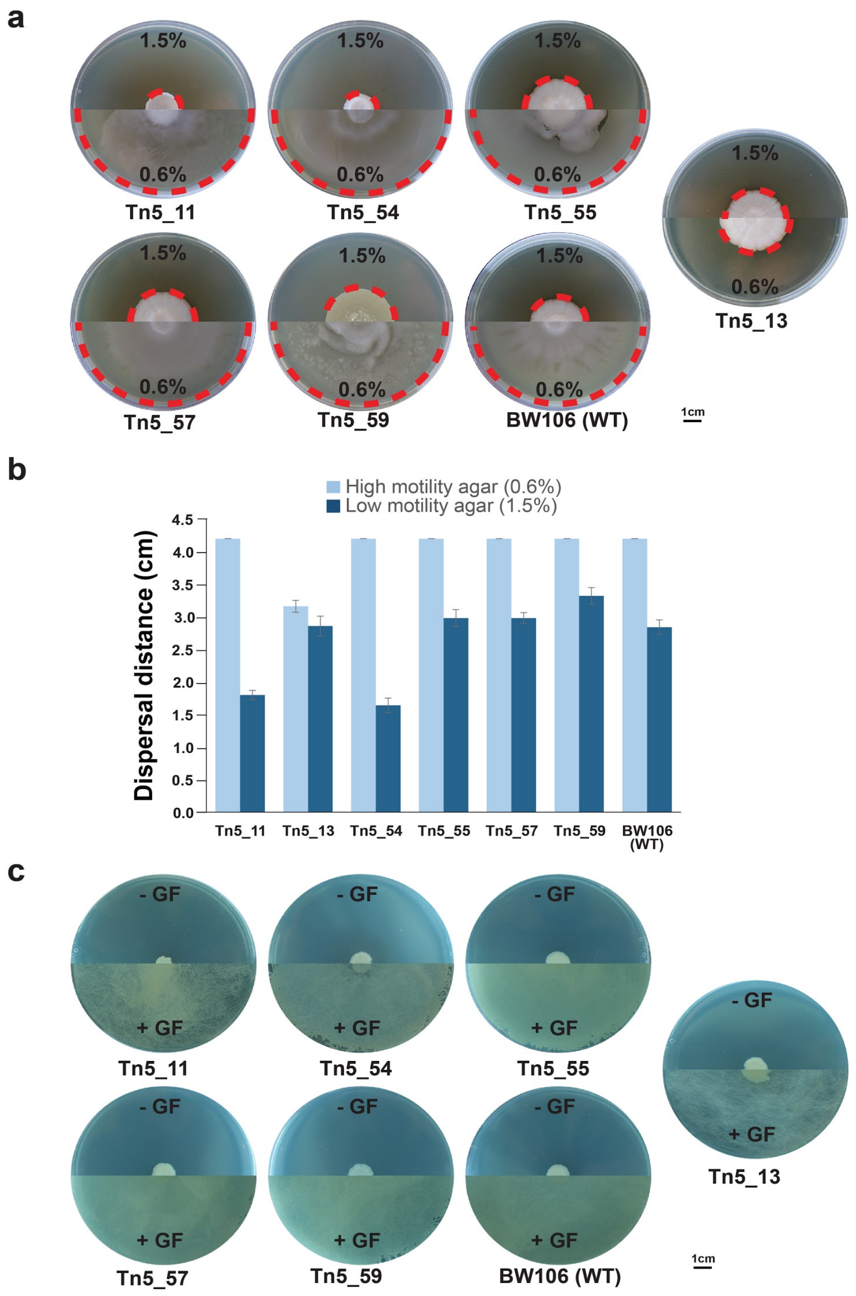

Supplementary Figure 5: Motility and growth on synthetic networks of Tn5 mutant strains. a Comparison of wild-type *Serratia* and putative mutants in low-motility conditions (1.5% agar, top half) and high-motility conditions (0.6% agar, bottom half). The edges of the bacterial colonies are outlined in red. Tn5_13, the mutant with a disruption in the *fliS* gene, is shown on the right. **b** Dispersal of the wild-type *Serratia* and the Tn5 mutants on high-motility agar (0.6%, light blue) and low-motility agar (1.5%, dark blue). Bars represent mean distance from center of spot to bacterial colony edge (+/- one standard deviation, n = 5) **c** Wild type *Serratia* and putative Tn5 mutants without (top) and with (bottom) a synthetic network made of glass fibers (GF). Tn5_13, shown on right, shows no dispersal on this artificial network, while the wild type *Serratia* and other mutants fully cover the glass fibers.

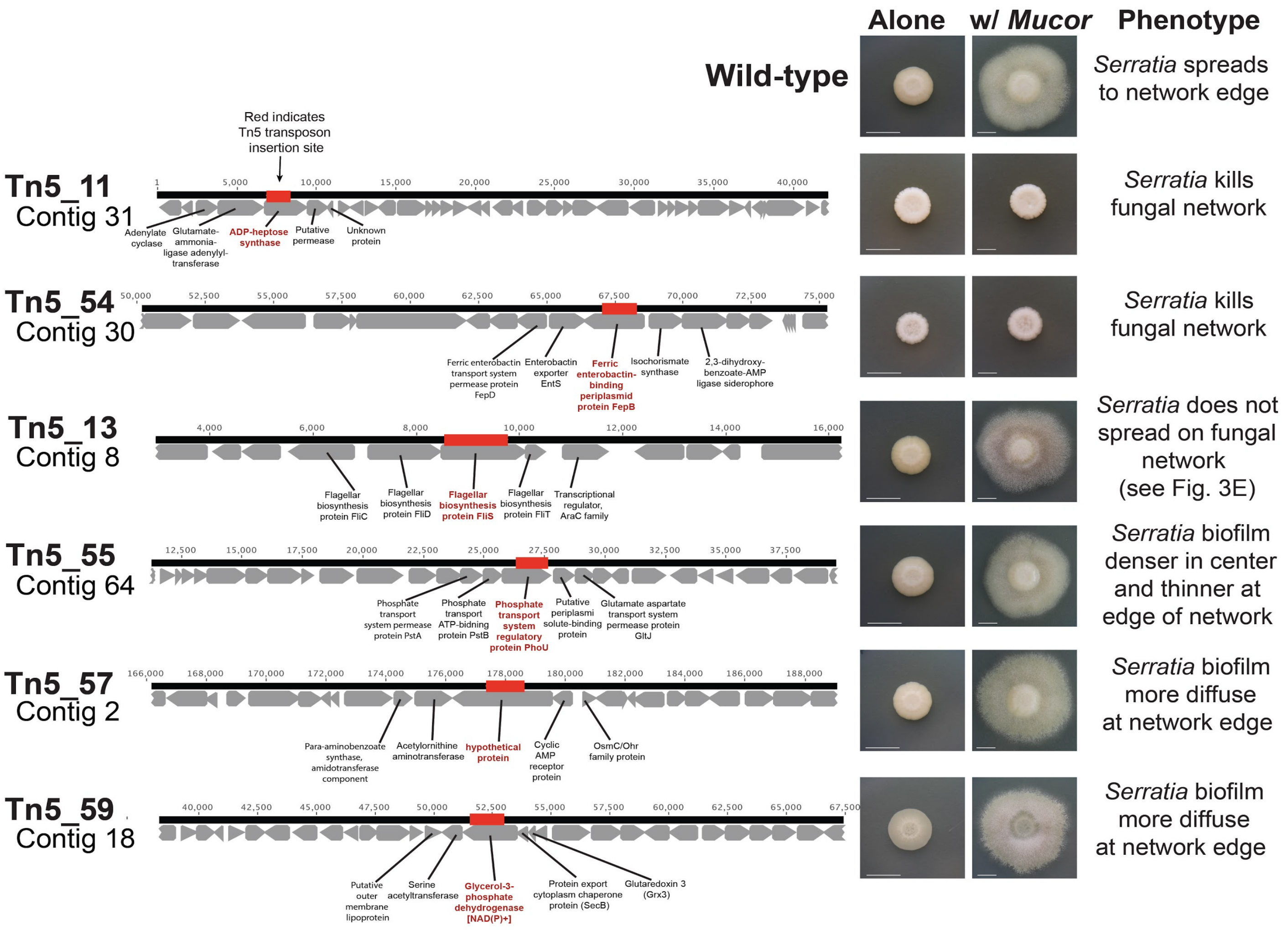

Supplementary Figure 6: Tn5 transposon insertion sites for various characterized mutants. Genomic context around insertion sites, as determined through whole-genome sequencing of mutant strains. Red indicates the position of the transposon insertion site. Photos show growth of *Serratia* spot alone (left) and with *Mucor* network (right) after 14 days of growth on PCAMS agar. White scale bar in photos indicates 1 cm. Phenotype describes the appearance of the *Serratia* biofilms on the *Mucor* networks.

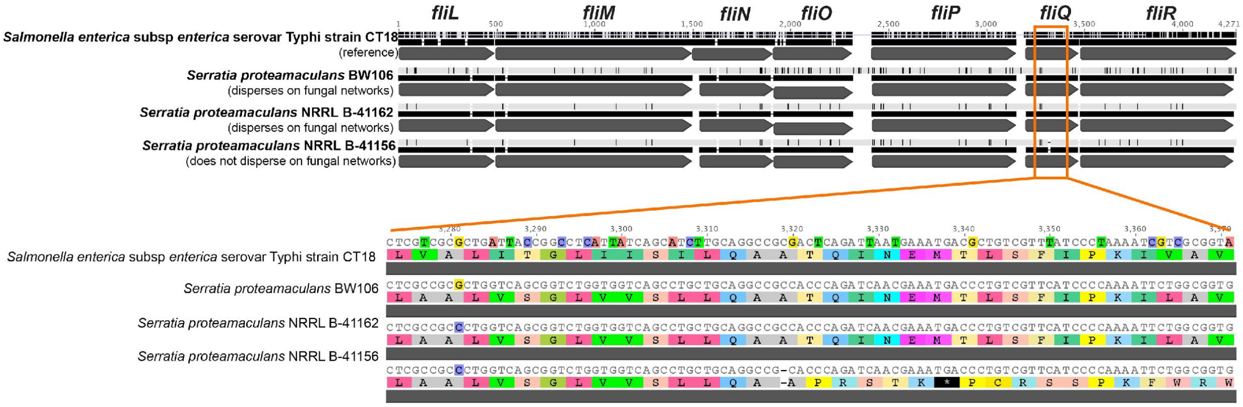

Supplementary Figure 7: Alignment of the *fliLMNOPQR* flagellar biosynthesis operon reveals a predicted loss-of-function mutation in a dispersal deficient strain. *Salmonella enterica* subsp. *enterica* Typhi strain CT18 as a reference sequence and three *Serratia proteamaculans* strains. Close-up section shows frameshift mutation in *fliQ* in *S. proteamaculans* strain B-41156.

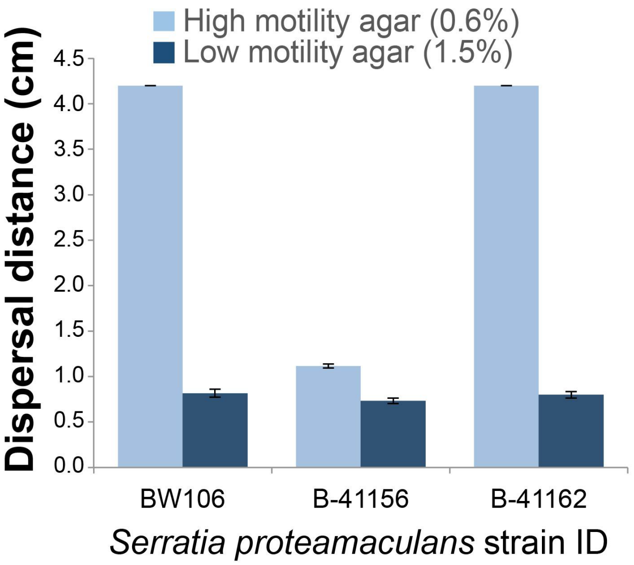

Supplementary Figure 8: Motility of flagellar mutant *S. proteamaculans* strain B-41156. Three different strains of *Serratia proteamaculans* (BW106, B-41156, and B-41162) were spotted on high-motility agar (0.6%, light blue) and low-motility agar (1.5%, dark blue). The *fliQ* mutant *S. proteamaculans* strain B-41156 has very limited motility compared to the other two strains. Bars represent mean distance from center of spot to bacterial colony edge (+/- one standard deviation, n = 3)

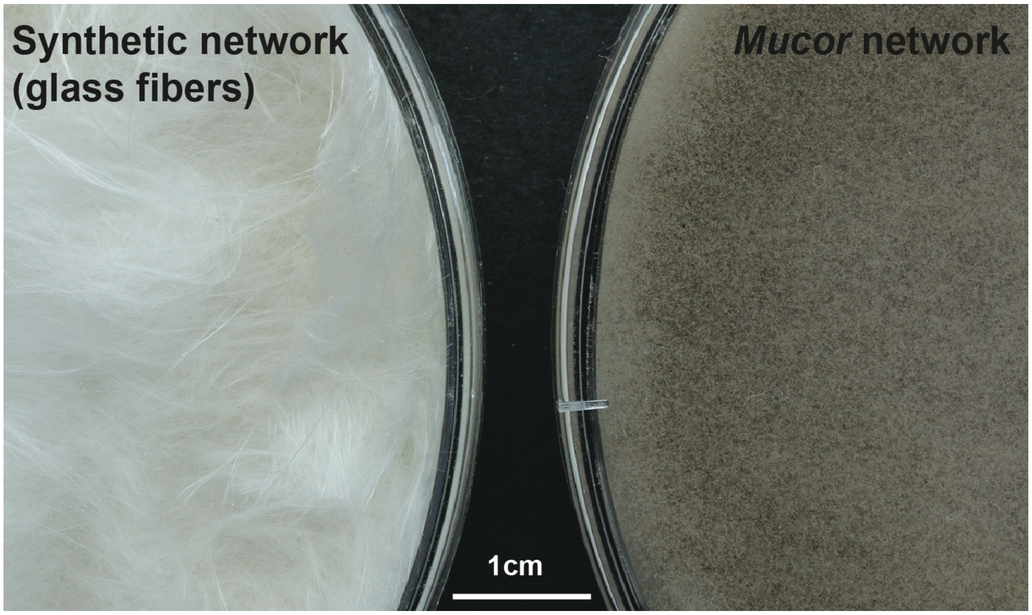

Supplementary Figure 9: Synthetic glass networks used to mimic fungal networks in community experiments. Shown on the left are glass fibers placed on the surface of cheese curd agar. On the right is *Mucor* growing on the surface of cheese curd agar. The dark fuzziness is spore production by the fungus.

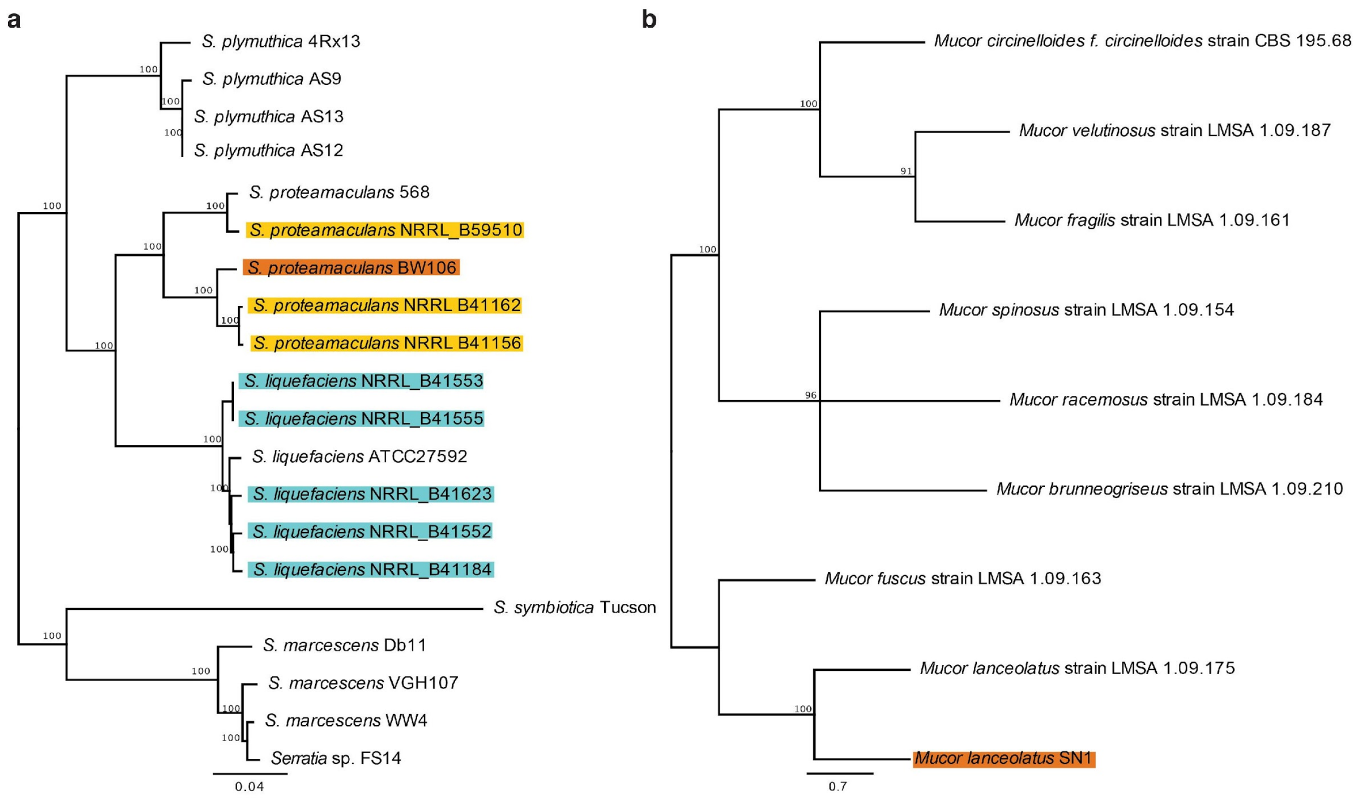

Supplementary Figure 10: Phylogenies of *Serratia* and *Mucor*, demonstrating relationships of strains used in study with previously characterized strains and species. a Maximum likelihood phylogeny of *Serratia* species and strains based on alignment of all single-copy genes identified from whole genome sequences. Reddish-orange highlighting indicates the cheese isolate (BW106) used throughout this study, yellow indicates strains of *S. proteamaculans*, and blue indicates strains of *S. liquefaciens*. **b** Maximum likelihood phylogeny of *Mucor* based on 18S rRNA, 28S rRNA, ITS, and rpb1 sequences from *Mucor* isolated from cheese and other environments. *Mucor lanceolatus* strain SN1 is the strain used throughout this paper.

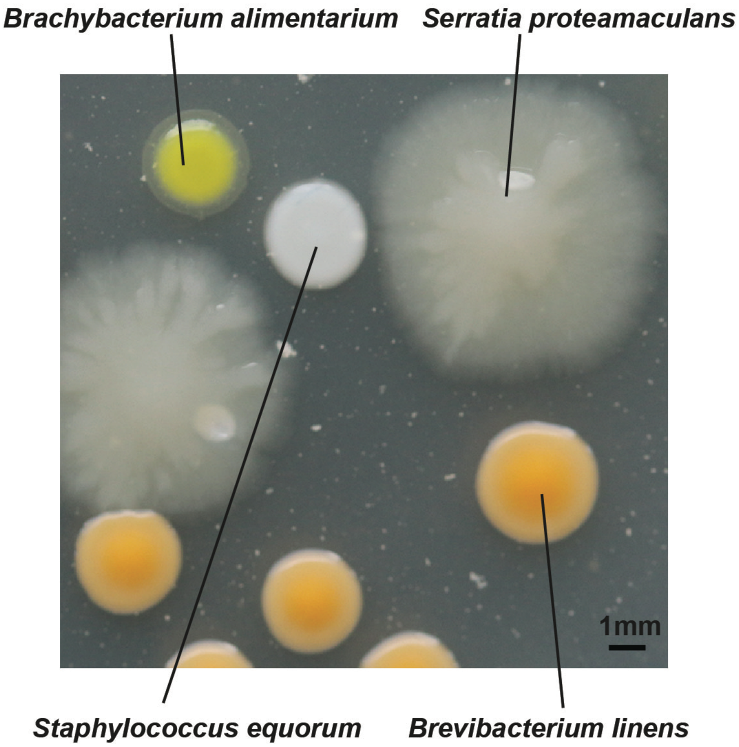

Supplementary Figure 11: Colonies of bacterial species used in experimental cheese rind communities. Each species can be easily distinguished by colony morphology on PCAMS agar.

Supplementary Table 1: Strains of *Serratia* and other microbes used throughout paper

StrainID#	Species	Isolation Source	NCBI Biosample #
**Bacteria Used in Experiments**			
BW106	*Serratia proteamaculans*	cheese rind, France	SAMN05449653
B-41156	*Serratia proteamaculans*	ground beef, USA (NRRL)	SAMN06055350
B-41162	*Serratia proteamaculans*	ground beef, USA (NRRL)	SAMN06055351
B-59510	*Serratia proteamaculans*	unknown (NRRL)	SAMN06055357
B-41184	*Serratia liquefaciens*	asparagus, USA (NRRL)	SAMN06055352
B-41552	*Serratia liquefaciens*	ground beef, USA (NRRL)	SAMN06055353
B-41553	*Serratia liquefaciens*	ground beef, USA (NRRL)	SAMN06055354
B-41555	*Serratia liquefaciens*	ground beef, USA (NRRL)	SAMN06055355
B-41623	*Serratia liquefaciens*	raw shrimp, USA (NRRL)	SAMN06055356
**Additional Bacteria Used in Comparative Genomics**			
4Rx13	*Serratia plymuthica*	roots of a potato plant	SAMN02603255
AS9	*Serratia plymuthica*	rapeseed roots	SAMN00713621
AS13	*Serratia plymuthica*	rapeseed roots	SAMN00713631
AS12	*Serratia plymuthica*	rapeseed roots	SAMN00713623
568	*Serratia proteamaculans*	roots of *Populus trichocarpa*	SAMN02598393
ATCC 27592	*Serratia liquefaciens*	unknown	SAMN02604177
Tuscon	*Serratia symbiotica*	aphid	SAMEA2581874
Db11	*Serratia marcescens*	moribund fly	SAMEA3138834
VGH107	*Serratia marcescens*	snakebite wound	SAMN02470620
WW4	*Serratia marcescens*	paper machine aggregates	SAMN02602965
FS14	*Serratia* sp.	*Atractylodes macrocephala*	SAMN03081466
**Bacteria Used in Community Experiments**			
JB425	*Providencia sp.*	cheese rind, Vermont, USA	---
JB418	*Pseudomonas sp.*	cheese rind, Vermont, USA	---
JB378	*Halomonas sp.*	cheese rind, Vermont, USA	---
JB37	*Vibrio sp.*	cheese rind, Vermont, USA	---
JB349	*Vibrio sp.*	cheese rind, Vermont, USA	---
JB196	*Psychrobacter sp.*	cheese rind, Vermont, USA	---
BP626-3	*Psychrobacter sp.*	cheese rind, Vermont, USA	---
JB193	*Psychrobacter sp.*	cheese rind, Vermont, USA	---
JB232	*Hafnia sp.*	cheese rind, Vermont, USA	---
BC10	*Staphylococcus xylosus*	cheese rind, Vermont, USA	---
BC9	*Staphylococcus equorum*	cheese rind, Vermont, USA	---
BC4	*Staphylococcus saprophyticus*	cheese rind, Vermont, USA	---
JB262	*Leucobacter sp.*	cheese rind, Vermont, USA	---
JB182.1	*Arthrobacter sp.*	cheese rind, Vermont, USA	---
JB111	*Arthrobacter sp.*	cheese rind, Vermont, USA	---
JB110	*Microbacterium sp.*	cheese rind, Vermont, USA	---
JB113	*Brachybacterium sp.*	cheese rind, Vermont, USA	---
JB9	*Brachybacterium sp.*	cheese rind, Vermont, USA	---
JB7	*Brachybacterium alimentarium*	cheese rind, Vermont, USA	---
JB11	*Brachybacterium sp.*	cheese rind, Vermont, USA	---
JB5	*Brevibacterium linens*	cheese rind, Vermont, USA	---
JB4	*Corynebacterium sp.*	cheese rind, Vermont, USA	---
**Fungi**			
SN1	*Mucor lanceolatus*	cheese rind, France	SAMN06042488
BW_12	*Penicillium* sp.	cheese rind, Vermont, USA	---
BW_242A	*Galactomyces geotrichum*	cheese rind, Vermont, USA	---
