# Supplementary material for: Fungal networks shape dynamics of bacterial dispersal and community assembly in cheese rind microbiomes
Source: Nat Commun. 2018 Jan 23;9:336. doi: 10.1038/s41467-017-02522-z (PMC5780524; doi:10.1038/s41467-017-02522-z)
Supplement: Supplementary file 3 — Description of Additional Supplementary Files [file 41467_2017_2522_MOESM3_ESM.pdf]

Description of Additional Supplementary Files

File Name: Supplementary Data 1
Description: Differentially expressed genes of *Serratia proteamaculans* BW106 when grown alone and on *Mucor* networks identified through RNA-seq. Highlighted in red are genes that had lower expression with *Serratia* was grown on *Mucor* networks. Highlighted in green are genes with higher expression when *Serratia* was grown on *Mucor* networks.

File Name: Supplementary Data 2
Description: Presence of accessory genes across three closely-related *Serratia proteamaculans* strains. Absence of gene family indicated with "-"

File Name: Supplementary Movie 1
Description: **Cells of *Serratia proteamaculans* swimming on fungal networks of *Mucor lanceolatus***. There is a faint but noticeable opacity difference between the liquid layer surrounding the hyphae and the background. Video was captured at 400X magnification.

File Name: Supplementary Movie 2
Description: ***Serratia* "pioneer" cells making initial contact with *Mucor*.** Two fungal hyphae are incoming from the left side of the field of view. One colony of *Serratia*, at slightly left of center, grows alongside the lower hypha. At 0:02, a single cell separates from the otherwise non-motile bacterial colony and begins to swim along the fungal hypha (indicated with red arrow). Video was captured at 400X magnification.

File Name: Supplementary Movie 3
Description: **Another example of a *Serratia* "pioneer" cell colonizing a *Mucor* hypha.** A pioneer cell (indicated with red arrow) that had previously separated from the stagnant bacterial colony around the tip of the hypha. This cell becomes obscured by the hypha before returning to the field of view. Video was captured at 400X magnification.
